# Remote Symptom Monitoring of Patients With Cancer Undergoing Radiation Therapy

**DOI:** 10.7759/cureus.29734

**Published:** 2022-09-29

**Authors:** Ryan Wahidi, Rachel Mintz, Temitope Agabalogun, Lindsey Mayer, Shahed Badiyan, Matthew B Spraker

**Affiliations:** 1 Radiation Oncology, Washington University School of Medicine, St. Louis, USA

**Keywords:** patient generated health data, neoplasm, short message service, radiotherapy, symptom assessment

## Abstract

The goal of the study was to develop and test an automated short message service (SMS) and web service platform using CareSignal for remote symptom monitoring in a diverse population of patients with cancer. Twenty-eight patients with cancer undergoing radiotherapy were recruited at the start of their treatment regimen. Patients received a weekly SMS symptom survey to assess the severity of the side effects they experienced from treatment. An assessment of patient perceptions of the system in terms of patient-provider communication, feasibility, and overall satisfaction was conducted, finding overall good compliance in a sick patient population and patient willingness to engage with the software in the future.

## Introduction

Digital health tools represent a range of hardware and software solutions designed to collect data and improve patient care, often in real time. Advancements in digital health tools have led to symptom monitoring systems such as web-based platforms that are both intuitive for patients to use and allow for the collection of sophisticated patient wellness measures.

Remote patient monitoring using digital health tools may be beneficial for patients with cancer receiving multimodal treatments such as radiation and chemotherapy due to the increased risk of acute and late toxic effects compared with those treated with less intense therapy. Also, it may be difficult for patients to easily recall symptoms they experienced between clinical encounters.

Studies have found that the use of web-based monitoring for patients with cancer is associated with improved overall survival and patient-reported quality of life [1-3]. Seminal studies by Basch et al. randomized patients undergoing chemotherapy to either receive standard care or standard care plus a web-based remote symptom reporting system [1,4]. The system allowed patients to submit weekly symptom reports, which were used in an alert system to notify providers of symptoms. The study found that patients who used the reporting system had improved patient-reported quality of life scores, fewer emergency room visits, and improved overall survival compared with those that did not. Further, patients who participated in remote symptom monitoring tolerated chemotherapy longer than those with standard care. Another study found that symptom monitoring protocols following cancer surgery reduced severe symptoms and improved patient performance status [5]. Similarly, remote patient surveillance after treatment for non-small-cell lung cancer resulted in improved patient outcomes [3].

One hypothesis for how symptom monitoring systems improve survival is that they allow teams to respond earlier and more efficiently to developing toxicities compared with routine surveillance protocols. This is supported by prior work that randomized patients undergoing chemotherapy to use symptom monitoring software with or without symptom care at home protocol including automated symptom self-management coaching and nurse practitioner follow-up [6]. Patients using Symptom Care at Home (SCH) group had a significant reduction in symptom burden (57%), reduction in days with severe (67%) or moderate (39%) symptoms, and reduction in all symptom scores except diarrhea compared with the non-symptom care at home group. This dramatic improvement in symptom control associated with the use of symptom monitoring systems may explain the reduced emergency room visits and improved overall survival observed in other studies.

These remote monitoring systems have been well studied in patients with cancer undergoing standard chemotherapy regimens but not radiotherapy, an increasingly prevalent cancer treatment. Definitive radiotherapy and concurrent chemoradiation therapy may be curative for patients with a variety of malignancies. Unfortunately, these treatments can be associated with high toxicity. A recent national trial of definitive chemoradiation therapy for non-small-cell lung cancer observed that 34%, 24%, and 22% of patients experienced pneumonitis, fatigue, and dyspnea, respectively [7]. Previous research by Lapen et al. has explored the use of questions from the patient-reported outcomes version of the common terminology criteria for adverse events (PRO-CTCAE) for remote symptom monitoring in patients receiving radiation for breast cancers and highlights the potential importance of remote monitoring as symptoms may develop as late as two weeks after treatment encounters [8]. There is significant interest in the use of short message service (SMS)-text-based symptom monitoring to reach a wider range of patients, as the use of more financially burdensome smartphones and computers is avoided [9-11].

This study sought to explore the acceptability of remote patient symptom monitoring in a diverse patient population, including those with thoracic malignancies or extremity/chest wall sarcoma malignancies undergoing multimodal therapy. CareSignal, a Health Insurance Portability and Accountability Act (HIPAA)-compliant SMS-text-based service, connects patients to clinicians via a web-based portal and allows for sophisticated monitoring of a wide range of symptoms. We used the CareSignal software and the PRO-CTCAE patient-reported measurement outcome system to design a low-resource symptom monitoring system for this patient population.

This prospective observational pilot study assessed the acceptability and user compliance of the remote symptom monitoring system in patients with thoracic malignancies or extremity/chest wall sarcomas undergoing radiation or chemoradiation.

## Materials and methods

**Figure 1:**
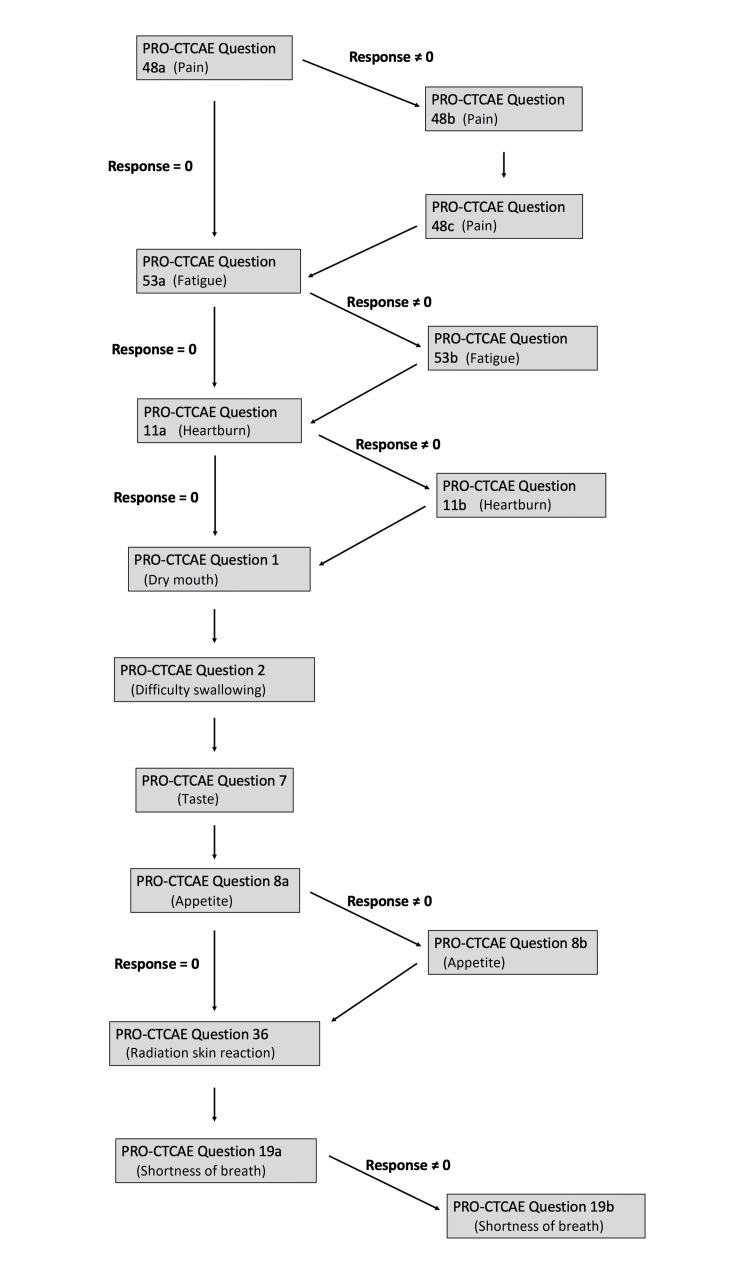
A sample flow of questions was presented to patients with non-small-cell lung cancer. Questions presented to the user depended on answers to previous questions. Responses to these questions were automatically recorded to a HIPAA-compliant online database. HIPAA: Health Insurance Portability and Accountability Act

This observational pilot study tested the acceptability of continuous symptom monitoring using PRO-CTCAE and the Epharmix Intervention Builder provided by CareSignal (St. Louis, MO). The CareSignal platform is a HIPAA-compliant system, and the study was approved by the Institutional Review Board (IRB) of Washington University School of Medicine. This service allows for linked question lists to be programmed and sent to patients via SMS-based text messaging to collect patient-reported data. Patients received text messages on a predetermined schedule, and responses were collected and stored for clinician use. Our intervention was programmed to send selected questions from the patient-reported outcomes version of the common terminology criteria for adverse events (PRO-CTCAE, https://healthcaredelivery.cancer.gov/pro-ctcae/) to patients via SMS at baseline, once per week during treatment, and for 30 days follow-up. Patients received one of three specific question lists based on whether they received thoracic radiation with chemotherapy, thoracic radiation without chemotherapy, or radiation outside the thorax. Question lists followed a branched logic in which follow-up questions depended on patient answers. An example of this logic is demonstrated in Figure 1. Complete question lists and branching logic for severity and interference questions are available in the figures in the Appendices.

Eligible patients were 18 years or older and planning to undergo radiotherapy for any cancer type for two or more fractions with or without chemotherapy. Enrollment was limited to a single clinical service within the department. The clinical care team for all patients consisted of one radiation oncologist and one nurse coordinator with or without a resident physician. The care team met with patients weekly during active treatment, as well as a 30-day follow-up. The care team was encouraged to remind patients to respond to text messages but did not have access to the data for assessment of the responses in the clinic. Enrolled patients participated in one training session during their initial visit where they entered baseline responses to the selected questions. Throughout treatment and follow-up, participants were asked to fill out the CareSignal questionnaire weekly. A complete response was defined as a numerical answer to each question asked. To calculate response rates, an incomplete response was treated as no response.

After completion of the final symptom survey, patients were invited to complete an additional survey regarding their experience with the CareSignal platform. The survey consisted of six three-point Likert scale items to assess the acceptability of integrating CareSignal into clinical practice (figures in Appendices).

A composite score was used to quantify each symptom with a single value to facilitate reporting and interpretation. In brief, each symptom receives a score of 0-3; symptoms assessed by multiple questions, such as pain, are granted a single composite score following the table presented in Basch et al. [12]. Detailed methodology and justification for this scoring system are likewise discussed by Basch et al. [12].

The primary endpoint was whether successful implementation was achieved. Successful implementation was defined as follows: 75% of enrolled patients completed at least 50% of all invited symptom surveys. To be considered a complete symptom survey response, at least 50% of questions needed to be answered in a given survey.

## Results

A total of 28 patients enrolled in the study from January 2020 to November 2020. One patient did not ultimately receive treatment but did enter responses in CareSignal, so that data was included in the analysis of acceptability. Patient and disease characteristics are shown in Table 1. There were 13 patients in the thoracic radiotherapy alone group and five patients in the thoracic chemoradiotherapy group. Ten patients had non-thoracic malignancies treated with radiotherapy. The median patient age was 65.2 years (range: 38-90 years). Most patients received palliative radiotherapy (70%), and the median number of fractions delivered was 5 (range: 5-33).

**Table 1:** Demographics of patients included in this study.

Demographic		Thoracic chemoradiotherapy (N=5)	Thoracic radiotherapy alone (N=13)	Non-thoracic malignancies treated with radiotherapy (N=10)	Overall (N=28)
Age (years)	Mean (SD)	69.2 (9.0)	61.8 (10.3)	67.6 (12.4)	65.2 (11.0)
	Median (min., max.)	67.6 (60.6, 84.1)	63.3 (40.4, 77.0)	64.2 (51.5, 91.2)	64.2 (40.4, 91.2)
Gender	Female	3 (60.0%)	6 (46.2%)	7 (70.0%)	16 (57.1%)
	Male	2 (40.0%)	7 (53.8%)	3 (30.0%)	12 (42.9%)
Race	Black or African American	0 (0%)	0 (0%)	2 (20.0%)	2 (7.1%)
	White	5 (100%)	13 (100%)	8 (80.0%)	26 (92.9%)
Ethnicity	Non-Hispanic	5 (100%)	13 (100%)	10 (100%)	28 (100%)

**Figure 2:**
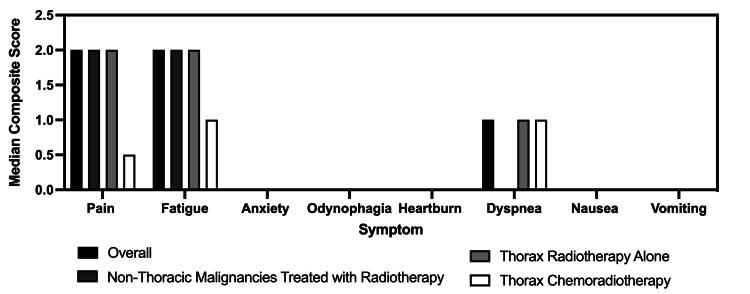
Median values for the severity of each symptom throughout treatment and follow-up across all patients and all surveys. Composite scores are reported, ranging from 0 to 3, 3 being the most severe.

For the SMS symptom surveys, complete response rates for each subgroup were calculated as the average of each individual’s response rate and were as follows: 34% (range: 0-71%) for thoracic chemoradiotherapy, 57% (range: 0-100%) for thoracic radiotherapy alone, and 76% (range: 14-100%) for non-thoracic malignancies with radiotherapy. The overall response rate across all groups was 60%. There was a subgroup of five individuals who provided a baseline response but did not respond to any additional questionnaires. If this group is excluded, the response rate for thoracic chemoradiotherapy was 50%, thoracic radiotherapy alone was 84%, and non-thoracic malignancies with radiotherapy was 83%, with an overall response rate of 79%. Median PRO-CTCAE scores for these categories are presented in Figure 2.

Surveys sent at the conclusion of the study were filled and returned by 11 of the 28 patients. Seventy-three percent (8/11) of respondents reported the SMS messaging system was easy to use, and 55% (5/11) noted that they would like to use it again in the future. Patients non-compliant with the SMS symptom surveys were not excluded from these survey responses.

## Discussion

Since the coronavirus disease 2019 (COVID-19) pandemic use of remote monitoring infrastructure, such as telemedicine, in cancer care has rapidly increased in prevalence [13]. Remote symptom monitoring solutions are starting to be explored in radiation oncology. This prospective pilot study evaluated SMS messaging and PRO-CTCAE for remote symptom monitoring in patients undergoing radiotherapy or multimodal treatment for thoracic and other cancers. An important feature of this implementation is that SMS messaging is broadly available and does not require a smartphone or computer; during recruitment, no patients were excluded due to a lack of technological access. Also, because standardized PRO-CTCAE items were used rather than proprietary questions, our findings can be more readily generalized [14].

The study focused on the acceptability of the system defined by the symptom survey response rate. While the study did not meet its primary endpoint of 75% compliance, fair compliance was achieved in this diverse population that included patients who are elderly as well as those undergoing treatment with chemoradiation or palliative radiotherapy. The limitations patients face due to the degree of symptoms from their treatment may present a barrier to adopting and interfacing with a digital health system [15]. Despite these barriers, Basch et al. found that such an older, more symptomatic, “computer-inexperienced” group derived the greatest benefit from the symptom monitoring intervention [12].

Several aspects of the implementation may have limited acceptability. The clinical team did not have access to patient responses in the clinic; real-time feedback from patients may improve the response rate. As suggested by the data, different treatment regimens, such as combined chemoradiotherapy may impact a patient’s compliance with this interface, and as such the heterogenous set of cancers and treatments in this study provides a limitation to assessing acceptability. Also, use was limited to a single clinical service within a larger department, so many staff in the clinic such as receptionists and therapists were not aware of the intervention. Implementation of the symptom monitoring system into the usual workflows used across all clinicians, staff, and patients for a department may increase compliance. An automated reminder system for patients who do not complete a questionnaire may also help. Patients reported that the SMS system was easy to use, and most would like to include it in their future care.

## Conclusions

The results of this study indicate that an SMS-based system may provide an effective means to remotely monitor symptoms of patients undergoing weekly radiotherapy. The intensity of treatment, such as combined chemoradiotherapy, may impact the response rates of individuals. Achieving a more robust level of compliance to weekly symptom surveys may require additional methods of engaging patients, such as discussing results in the clinic and a patient reminder system. Future studies may explore further integrated remote monitoring within the clinical environment and physician-patient interface and analyze symptom trends among a larger number of patients.

## Figures and Tables

**Figure 3:**
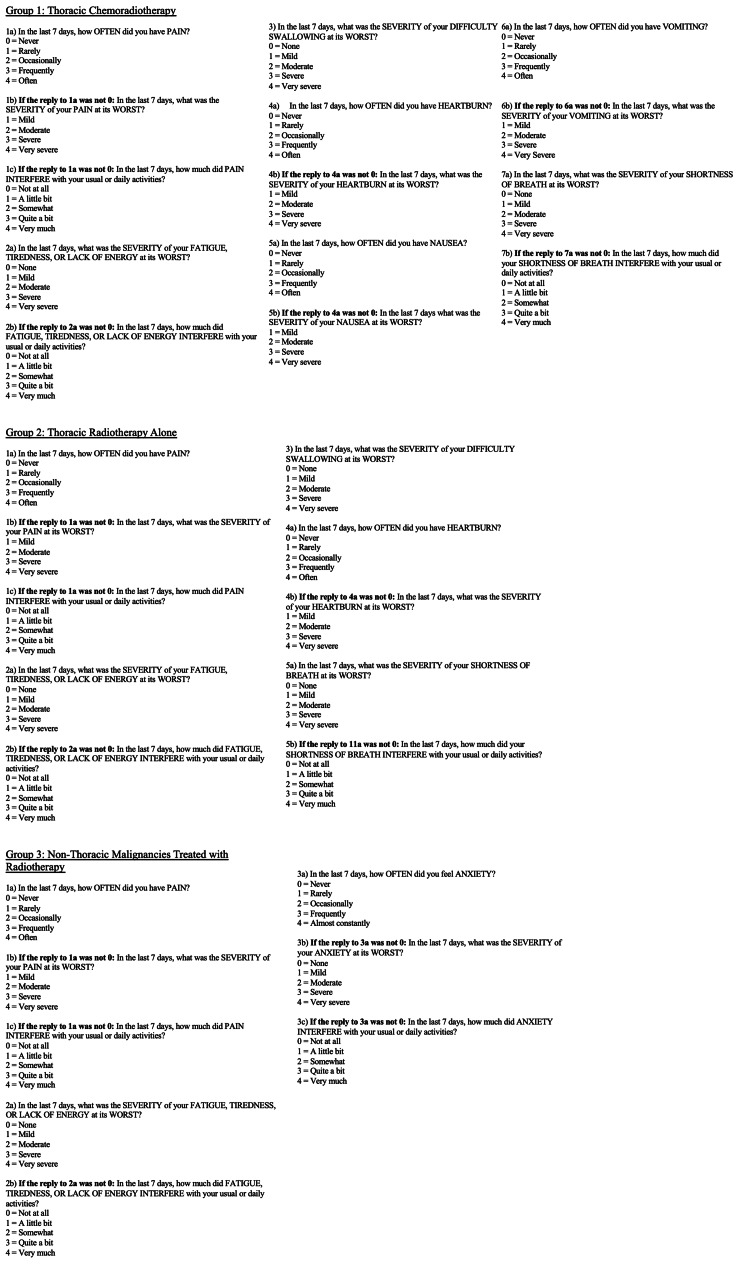
Aggregate list of questions sent to patients in the study.

**Figure 4:**
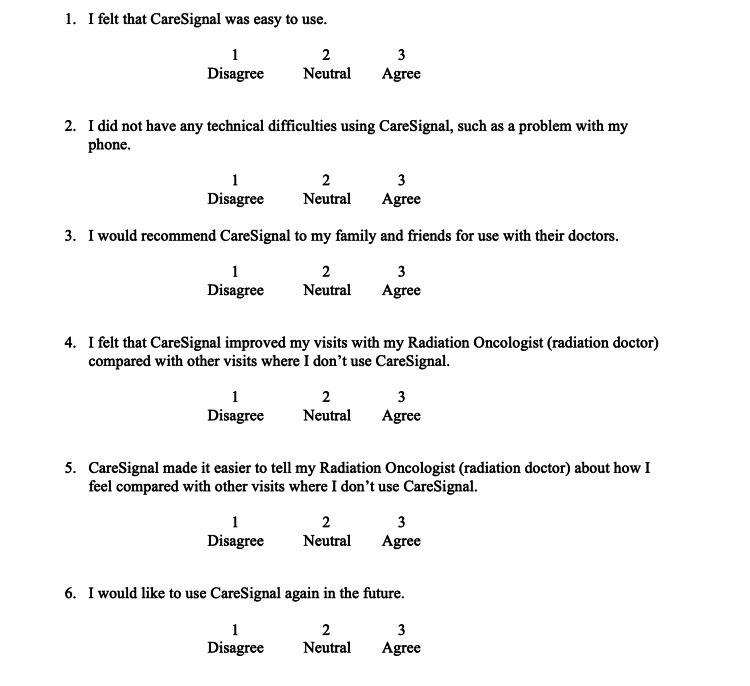
Survey sent at the completion of symptom monitoring.
